# The Choice Between Definitive Radiotherapy and Surgery for Cervical Cancer With Different Histology in Current Clinical Practice

**DOI:** 10.1002/kjm2.70272

**Published:** 2026-08-20

**Authors:** Yu‐Lun Wu, Chen‐Wei Ho, Wen‐Shiung Liou, Chun‐Hao Yin, Jin‐Shuen Chen, Yao‐Shen Chen, Yu‐Wei Lin, Ju‐Chun Chien

**Affiliations:** ^1^ Department of Radiation Oncology Kaohsiung Veterans General Hospital Kaohsiung Taiwan; ^2^ Department of Radiology Kaohsiung Veterans General Hospital Kaohsiung Taiwan; ^3^ Department of Obstetrics and Gynecology Kaohsiung Veterans General Hospital Kaohsiung Taiwan; ^4^ Department of Medical Education and Research Kaohsiung Veterans General Hospital Kaohsiung Taiwan; ^5^ Department of Business Management National Sun Yat‐Sen University Kaohsiung Taiwan; ^6^ Department of Administration Kaohsiung Veterans General Hospital Kaohsiung Taiwan; ^7^ School of Medicine College of Medicine, National Sun Yat‐Sen University Kaohsiung Taiwan; ^8^ Office of Research and Development With International Affairs Tajen University Pingtung Taiwan

**Keywords:** adenocarcinoma, cervical cancer, radiotherapy, surgery

## Abstract

For cervical cancer, the choice between definitive radiotherapy (RT) and surgery has long been a topic of debate. Clinical factors, including histology and tumor burden, have been examined in prior studies. Given recent advances in RT and the incorporation of concurrent chemotherapy, our study seeks to assess the optimal treatment modality for cervical cancer in current clinical practice. The cancer registry database of a medical center was screened for cervical cancer patients. Patients receiving curative intent RT or surgery were included. Clinicopathological factors and treatment outcomes were retrospectively collected. The disease‐free survival (DFS) and overall survival (OS) were analyzed using Cox regression and Kaplan–Meier analyses. From 2007 to 2021, 369 squamous cell carcinoma (SCC) and 108 adenocarcinoma (AC) cases were enrolled. AC had an increasing proportion over time and was associated with worse survival. Comparing RT to surgery, RT was associated with inferior survival in both early‐stage AC (DFS, HR: 8.29, *p* < 0.001, 95% CI: 3.21–21.42) and SCC group (DFS, HR: 4.01, *p* < 0.001, 95% CI: 1.99–8.08). The difference was more robust in those with tumors smaller than 4 cm. For advanced‐stage disease, the inferiority of RT was observed in the SCC (DFS, HR: 1.85, *p* = 0.020, 95% CI: 1.10–3.11), but not AC group. There was no statistically significant intergroup difference observed between the AC and SCC groups regarding DFS of RT versus surgery. In our analysis, AC was an independent poor prognostic factor. Despite the addition of concurrent chemotherapy and advanced RT techniques, surgery‐based treatment was still associated with superior survival for early‐stage cervical cancer.

## 1 Introduction

Cervical cancer is one of the most common malignancies threatening women and remains a significant cause of female mortality worldwide. According to the Global Burden of Disease Study, more than 600,000 cases were diagnosed in 2021, with an estimated 300,000 deaths [1].

Squamous cell carcinoma (SCC) and adenocarcinoma (AC) are two of the most common histological subtypes for cervical cancer. They differ markedly in their etiology, screening sensitivity, prognosis, and treatment response. The randomized controlled trial conducted by Landoni et al. targeting International Federation of Gynecology and Obstetrics (FIGO) Stage IB–IIA cervical cancer stated that radiotherapy (RT) led to a superior overall survival (OS) in the SCC subgroup [2, 3]. On the other hand, radical hysterectomy was more effective for the AC subgroup. However, this trial employed outdated RT techniques and did not incorporate concurrent chemotherapy, which is now the standard of care proven to improve survival [4, 5].

The choice between surgery‐based treatment and definitive RT for cervical cancer remains a subject of ongoing debate, balancing disease control and treatment toxicity. Definitive RT has been proposed for advanced cervical cancer to prevent multimodal treatment, which was stated to have similar disease control but with greater toxicity [4, 5, 6]. However, the rationale hasn't been evaluated directly in advanced disease, and it warrants careful evaluation in cervical AC given its intrinsic radioresistance [7, 8, 9]. A retrospective cohort study focusing on bulky cervical cancer reported a lower locoregional recurrence risk with radical hysterectomy in the high‐risk group, including AC or adenosquamous histology [10]. More than half of them have received adjuvant RT. If surgery could help overcome the radioresistance of AC, the disease control benefit might outweigh treatment‐related adverse effects from multimodel cancer therapy. Yet, literature comparing surgery and RT for advanced cervical cancer was sparse.

In the past decades, the addition of concurrent chemotherapy and the shift from four‐field box technique to intensity‐modulated radiation therapy (IMRT) have greatly influenced the treatment outcome of RT. A global increase in cervical AC incidence has also been noted, particularly in Western countries [11, 12, 13]. Accordingly, it is crucial to refine treatment strategies based on modern techniques with histological characteristics. Therefore, our study aimed to evaluate current clinical practice and assess treatment modalities for cervical cancer across different histology groups in current clinical practice.

## 2 Methods

### 2.1 Study Cohort and Data Collection

The study was conducted in accordance with the Declaration of Helsinki and was approved by the Institutional Review Board of Kaohsiung Veterans General Hospital (KSVGH23‐CT12 18; date of approval: December 18, 2023). The requirement for patients' informed consent to participate was waived following approval by the Institutional Review Board of Kaohsiung Veterans General Hospital, since all clinical data were collected retrospectively from medical records and the institutional cancer registry database. The cancer registry database of one medical center was retrospectively screened for pathologically proven cervical cancer patients. In terms of histologic subtypes, only patients with SCC or AC, based on the ICD‐O‐3 morphology codes, were included. Patients with cervical malignancies other than SCC or AC, distant metastasis at diagnosis, a history of prior malignancy, or no curative local treatment were excluded. Medical records and imaging studies were reviewed to collect clinicopathologic factors, treatment information, and clinical outcomes. Patients were categorized into the surgery‐based treatment and the RT subgroups according to the primary curative‐intent local treatment modality. Early‐stage disease was defined as Stage IA–IIA, while advanced‐stage disease was defined as Stage IIB–IVA according to the Revised FIGO 2018 staging.

The primary outcome was disease‐free survival (DFS), defined as patients being alive without any disease‐related outcome, including persistent disease, local recurrence, regional failure, or metastasis. For RT subgroup, evidence of residual disease would only be recorded as persistent disease at least 3 months after completion of the RT. The secondary outcome was OS. These survival outcomes were calculated from the complete date of the primary curative‐intent local treatment. Sensitivity analyses with survival outcomes calculated from the diagnosis date were performed to evaluate the influence of time‐origin bias.

### 2.2 Statistical Analysis

The enrolled cohort was stratified by histology into SCC and the AC groups. Differences in baseline characteristics were assessed using the Student's *t*‐test for continuous variables and the chi‐squared test or Fisher's exact test for categorical variables. Survival outcomes were analyzed by the Kaplan–Meier analysis and the Cox regression test. Variables with distributional imbalance and known prognostic impact were considered in the multivariate analysis. Hazard ratios (HRs) and the 95% confidence intervals (CIs) were reported as relative risk estimates. To compare treatment results between the surgery‐based treatment and the RT subgroups, stratification and multivariate Cox regression were applied to address possible selection bias. Series of sensitivity analyses were performed with exclusion of patients who underwent conization or trachelectomy from the surgery‐based treatment group, and with further stratification of them into surgery alone and surgery with adjuvant treatment subgroups. All statistical analyses were performed using Statistical Analysis Software (SAS; version 9.4; SAS System for Windows) and the Statistical Package for Social Sciences, statistical software 20 (IBM Corp., Armonk, NY, USA). Statistical significance was set at a two‐tailed *p* < 0.05.

## 3 Results

From January 2007 to December 2021, 648 continuous cervical cancer cases from the cancer registration database were screened, among which 477 patients met the inclusion criteria and were enrolled in our original study cohort. In terms of histology, 369 (77.4%) patients had SCC, and 108 (22.6%) patients had AC. Although the incidence of cervical cancer decreased, cervical AC increased in proportion in more recent years and affected younger women (Table 1).

**TABLE 1:** Baseline characteristics of patients with cervical cancer.

Variable	All patients, *n* = 477 (%)	ADENO, *n* = 108 (%)	SCC, *n* = 369 (%)	*p*
Age at diagnosis, year	54.7 ± 13.0	51.2 ± 10.9	55.7 ± 13.3	0.001
< 65	372 (78.0)	95 (88.0)	277 (75.1)	0.004
≥ 65	105 (22.0)	13 (12.0)	92 (24.9)	
Date of initial diagnosis				0.012
2007–2014	306 (64.2)	58 (53.7)	248 (67.2)	
2015–2021	171 (35.8)	50 (46.3)	121 (32.8)	
Grade/differentiation^a^				< 0.001
Grade 1	26 (7.0)	20 (23.3)	6 (2.1)	
Grade 2	292 (78.5)	43 (50.0)	249 (87.1)	
Grade 3	54 (14.5)	23 (26.7)	31 (10.8)	
HPV status^a^				0.913
Negative	5 (9.6)	3 (10.0)	2 (09.1)	
Positive	47 (90.4)	27 (90.0)	20 (591.0)	
Clinical tumor size^a^				0.208
≤ 4 cm	287 (63.4)	69 (65.1)	218 (62.8)	
> 4 cm	166 (36.6)	37 (34.9)	129 (37.2)	
Clinical FIGO staging^b^				0.006
IA–IIA	324 (67.9)	85 (84.3)	239 (64.8)	
IIB–IVA	153 (32.1)	23 (15.7)	130 (35.2)	
Primary treatment modality				0.137
Surgery^c^	353 (74.0)	86 (79.6)	267 (72.4)	
Definitive RT	124 (26.0)	22 (20.4)	102 (27.6)	
Concurrent CT				0.174
With CT^d^	94 (75.8)	14 (63.6)	80 (78.4)	
Without CT	30 (24.2)	8 (36.4)	22 (21.6)	

Abbreviations: ADENO = adenocarcinoma; CT = chemotherapy; FIGO = International Federation of Gynecology and Obstetrics; RT = radiotherapy; SCC = squamous cell carcinoma.

^a^ Missing: grade/differentiation: 105; HPV status: 425; clinical tumor size: 24.

^b^ The clinical FIGO staging was based on the Revised FIGO stage 2018.

^c^ Surgical intervention including conization in *n* = 12, trachelectomy in *n* = 3, and hysterectomy in *n* = 337; missing *n* = 1.

^d^ Concurrent chemotherapy was applied according to GOG 120 with weekly cisplatin mainly.

In regard to the primary curative‐intent local treatment, surgery‐based treatment was performed for 353 patients, while 124 received definitive RT. The choice between treatment options was significantly associated with age, tumor size, and clinical stage, but not histological groups (Table S1). For those receiving primary surgery, 46.2% underwent further adjuvant RT or concurrent chemoradiotherapy (CCRT). The patients with parametrial invasion, positive surgical margin, and lymph node metastasis found after the definite surgery for cervical cancer would be offered adjuvant CCRT. Adjuvant RT would be provided for patients without the above‐mentioned major risk factors but meeting the Sedlis Criteria. In the definitive RT group, the standard external beam radiotherapy (EBRT) regimen consisted of 45 Gy in 25 fractions to the lymphatic basins at risk, with boost to 50 Gy in 25 fractions to the gross tumor, parametrium, and uterus. Concurrent chemotherapy was administered with four to five doses of weekly platinum. High‐dose‐rate intracavitary brachytherapy was delivered by Ir‐192 with 30 Gy in five fractions following EBRT. EBRT was performed with three‐dimensional conformal radiotherapy (3DCRT) in 34% and IMRT in 66%, with concurrent chemotherapy given in 75.8% of the patients. Image‐based intracavity brachytherapy was applied in 43.9%.

After the median follow‐up of 69.0 months (IQR: 42.7, 120.0 months), the 5‐year DFS and OS were 82.6% (95% CI: 78.3%–86.9%) and 89.0% (95% CI: 85.5%–92.5%) for FIGO Stage IA–IIA, and were 41.6% (95% CI: 33.6%–49.6%) and 56.4% (95% CI: 48.2%–64.6%) for Stage IIB–IVA. In the multivariate analysis, after adjusting for baseline distributional differences and known prognostic factors, histology remained a decisive prognostic factor for cervical cancer (Table 2). The SCC histology was associated with superior DFS (HR: 0.50, 95% CI: 0.32–0.76) and OS (HR: 0.44, 95% CI: 0.27–0.70) than AC. The prognostic value of histology might not be confounded by misclassification of clinical stage or other known prognostic factors, since the permanent pathology of those who underwent surgery revealed less frequent lymph‐vascular space invasion (LVSI) in AC. At the same time, no difference in pathological staging, tumor size, or depth of invasion (DOI) was observed (Table S2). AC could be taken as an independent poor prognostic factor for cervical cancer. On the other hand, surgery‐based treatment was associated with superior DFS and OS after multivariate analysis. Sensitivity analyses were performed with diagnosis date as Day 0 (Table S3) and with further stratification of the surgery‐based treatment group (Table S4). The results all remained consistent with the primary analyses.

**TABLE 2:** Comparison of survival outcome.

Variable	Overall survival		Disease‐free survival	
	Multivariate		Multivariate	
	HR (95% CI)	*p*	HR (95% CI)	*p*
Age at diagnosis, year				
< 65	Ref		Ref	
≥ 65	2.68 (1.85–3.87)	< 0.001	2.19 (1.57–3.06)	< 0.001
Date of initial diagnosis				
2007–2014	Ref		Ref	
2015–2021	0.45 (0.29–0.71)	< 0.001	0.58 (0.40–0.84)	0.003
Histology				
SCC	0.44 (0.27–0.70)	0.001	0.50 (0.32–0.76)	0.001
ADENO	Ref		Ref	
Differentiation				
Grade 1	Ref		Ref	
Grade 2	1.18 (0.48–2.90)	0.715	1.39 (0.58–3.35)	0.465
Grade 3	1.81 (0.72–4.58)	0.210	2.16 (0.87–5.39)	0.097
Clinical FIGO staging^a^				
IA–IIA	Ref		Ref	
IIB–IVA	1.91 (1.19–3.06)	0.007	2.48 (1.61–3.83)	< 0.001
Tumor size				
≤ 4 cm	Ref		Ref	
> 4 cm	2.20 (1.45–3.32)	< 0.001	1.76 (1.20–2.58)	0.004
Primary treatment modality				
Surgery	Ref		Ref	
Definitive RT	2.33 (1.50–3.62)	< 0.001	2.59 (1.71–3.72)	< 0.001

Abbreviations: ADENO = adenocarcinoma; CI = confidence interval; FIGO = International Federation of Gynecology and Obstetrics; HR = hazard ratio; RT = radiotherapy; SCC = squamous cell carcinoma.

^a^ The clinical FIGO staging was based on the Revise FIGO stage 2018.

**FIGURE 1:**
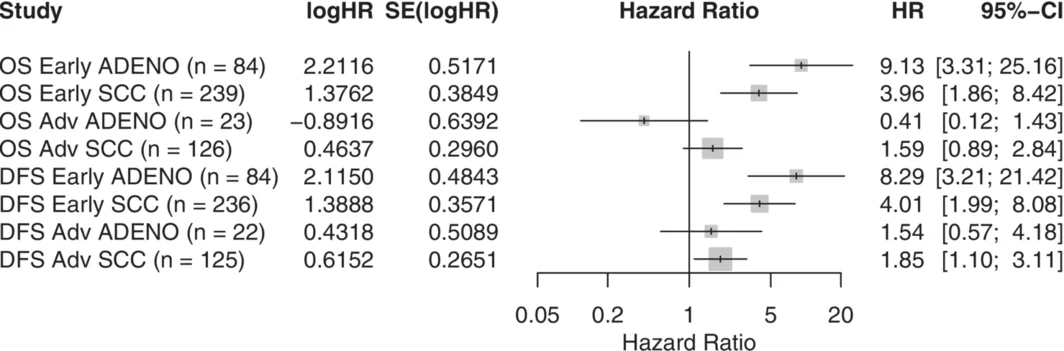
OS and DFS of each subgroup. The figure showed DFS and OS comparing treatment modalities for each subgroup, stratified by stage and histology. The surgical arm was taken as the reference group, and hazard ratios exceeding 1 corresponded to worse outcomes in the radiotherapy arm. Regarding treatment outcomes, 5 and 10 patients were excluded from the OS (*n* = 472) and DFS (*n* = 467) analyses, respectively, due to missing survival status and disease status data.

To eliminate the impact of important prognostic factors and to better reflect the clinical scenario, surgery‐based treatment and definitive RT were compared with stratification by histological type and clinical FIGO stage. The results were presented in Figure 1. For early‐stage cervical cancer, RT was associated with a significantly lower 5‐year DFS (AC, HR: 8.29, *p* < 0.001; SCC, HR: 4.01, *p* < 0.001) and 5‐year OS (AC, HR: 9.13, *p* < 0.001; SCC, HR: 3.96, *p* < 0.001), in both histology groups. The intergroup analysis showed no significant difference between SCC and AC subgroups (*p* = 0.20) (Figure 1). We further stratified early‐stage SCC and AC according to tumor size, and found that RT was still less effective in smaller tumors (≤ 4 cm) in terms of DFS (SCC: HR 6.38, *p* < 0.001；AC: HR 18.92, *p* < 0.001). In the early‐stage SCC group with tumor larger than 4 cm, there was a trend showing RT to be less effective, but no statistical significance was found (Table S5). The DFS of RT for the advanced‐stage SCC group was inferior to surgery (HR: 1.85, *p* = 0.020, 95% CI: 1.10–3.11), whereas OS did not differ significantly between the two treatment groups. For advanced‐stage AC, no difference was observed in DFS or OS between treatment modalities in this analysis.

## 4 Discussion

In our present study, which incorporated current treatment modalities, the DFS and OS of cervical cancer have steadily improved. The proportion of AC has been increasing, particularly in younger patients. In terms of survival outcomes, our study found that AC was associated with significantly poorer DFS and OS. For early‐stage disease, surgery‐based treatment was linked with superior survivals, especially in the case of AC. In advanced‐stage cervical AC, however, no significant difference in survival was observed between definitive RT and surgery.

### 4.1 Improved Survival With Modern Treatment Modalities

Compared with the 5‐year DFS of 74% and OS of 83% reported by Landoni et al. [3], our study revealed a superior survival outcome in early‐stage cervical cancer (5‐year DFS: 82.6%, 5‐year OS: 89.0%). The improvement was also observed over time within our cohort. In earlier clinical trials and FIGO staging system, cervical cancer staging relied primarily on pelvic examination rather than diagnostic imaging. With the application of advanced radiologic techniques, including magnetic resonance imaging (MRI) and positron emission tomography (PET), we are now identifying parametrial or nodal involvement in the earlier stage of the disease's development [14, 15]. Accordingly, reporting treatment outcomes based on the updated FIGO staging system and methods is crucial. Moreover, the advances in chemotherapy and RT techniques might also contribute to the improvement of cervical cancer treatment. Concurrent chemotherapy provides superior OS and DFS compared with RT alone [4, 5, 16]. The shift from four‐field box RT to IMRT has enabled more precise radiation delivery to the target region while reducing toxicity [17, 18, 19, 20]. Notably, the proportion of AC in our cohort was nearly twice that reported in the previous trial [3]. These developments in diagnostic methods, treatment approaches, and the evolving distribution of histologic subtypes over time highlight the need to reassess treatment strategies tailored to specific stages and histologic profiles.

### 4.2 AC: Raising Proportion and the Prognostic Impacts

The incidence of cervical AC has increased over time, particularly in Western countries [11, 12, 13]. In our cohort, the proportion of AC was significantly higher in patients diagnosed at younger ages and in more recent years. This phenomenon underscored the need to evaluate the influence of AC on prognosis and treatment strategies. Several studies have identified AC as an independent predictor of poorer survival [7, 8, 9, 10, 21, 22]. AC was also considered an independent poor prognostic factor in our analysis, which was not confounded by other known clinicopathological prognostic factors. The potential intrinsic radioresistance of AC has been highlighted in previous research [7, 8, 9]. The addition of concurrent chemotherapy as a radiosensitizer to either definitive or adjuvant RT has been considered the optimal approach in recent years [23]. Other studies have evaluated the benefit of radical surgery in more advanced stages of cervical AC [10, 24, 25, 26].

### 4.3 Role of Surgery in Early‐Stage Disease

For early‐stage cervical cancer, surgery was associated with superior DFS and OS compared with definitive RT in our cohort, both in the SCC and AC groups. The advantage of surgery was more robust in cervical tumors less than 4 cm. This result should be viewed with the awareness of potential selection bias arising from tumor burden and medical comorbidity. The definition of DFS events might also favor the surgical arm as gross residual disease could be detected earlier in the RT group. Despite these possible biases, our observations were consistent with previous literature. Landoni's trial indicated that the surgical treatment was comparable to RT when the cervical tumor was no larger than 3 cm [2, 3]. Two large retrospective cohort studies based on the Surveillance, Epidemiology, and End Results (SEER) database also demonstrated that surgery was the superior choice for early‐stage cervical cancer in terms of OS [27, 28]. Further stratified analyses revealed that the superiority of surgery over RT was more pronounced in tumors smaller than 4 or 6 cm and diminished with larger tumors [27, 28]. Based on current evidence, surgery‐based treatment was associated with better survival for early cervical cancer when compared to definitive RT. Before a further randomized control trial is available, however, these findings should be interpreted as an association rather than definitive evidence of treatment superiority.

Regarding the histological subtypes, there was no statistically significant inter‐subgroup difference found in the surgical benefit in our early‐stage cohort. Nevertheless, Landoni's trial demonstrated that treatment outcomes differed between surgery and RT according to histological type [2, 3]. A meta‐analysis was also interested in comparing surgery versus RT for patients with early‐stage AC [6]. Up to the present, the predicting value of histology in cervical cancer was not well established and not incorporated into the treatment guideline. In light of the increasing proportion of AC, further research would be warranted.

### 4.4 Role of Surgery in Advanced‐Stage Cervical AC

For advanced‐stage cervical cancer, an increasing trend of upfront surgery was demonstrated, particularly among those with AC [25]. This trend may reflect growing concerns regarding the suboptimal response of AC to RT. An analysis based on the SEER database reported that surgery combined with adjuvant RT conferred survival benefits in the advanced AC group compared to definitive RT, though it did not account for all relevant treatment variables such as concurrent chemotherapy or RT techniques [26]. In a recent cohort study comparing radical hysterectomy to definitive CCRT, surgery led to better recurrence‐free survival (75.2% vs. 59.6%, *p* = 0.036) and fewer local‐regional recurrence (11.6% vs. 26.3%, *p* = 0.023) in bulky Stage IB–IIA cervical cancer with high‐risk histology (i.e., AC or adenosquamous carcinoma) [10]. Another large cohort study from Japan revealed that surgery was associated with favorable 5‐year OS for Stage IB2–IIB cervical AC (HR = 1.419; 95% CI = 1.084–1.858; *p* = 0.011) [24]. With inclusion of more advanced disease, there was no survival benefit observed in patients with advanced‐stage AC receiving surgery in our analysis, despite potential biases favoring the surgical arm. The preference of surgery in AC could not be extrapolated to cervical cancer of any stage based on current evidence. In contrary to other subgroups, the HR of OS for advanced‐stage AC demonstrated a vague trend favoring RT. However, this phenomenon was not observed in DFS. Given the very limited sample size, this finding should be interpreted cautiously with insight of statistical uncertainty. Careful selection of advanced cervical AC for radical surgery is important to provide a more precise and personalized treatment. Other approaches, including the addition of immunotherapy, might warrant further evaluation to improve the treatment of advanced‐stage AC [29].

## 5 Limitation

This study has several limitations. First, the number of patients with advanced‐stage disease was relatively small, likely reflecting the impact of nationwide cervical cancer screening programs. This may have reduced the statistical power to detect differences in survival and increased the risk of Type II error. Therefore, the absence of statistically significant survival differences in the advanced‐stage analyses should not be interpreted as definitive evidence of equivalent treatment efficacy. Second, selection bias may persist given the retrospective nature of the study. Although efforts were made to reduce this bias by excluding patients with poor general health status, the potential for residual bias could not be entirely excluded. However, the interpretation of our main findings in the advanced‐stage AC group was unaffected. Third, treatment‐related adverse events were not assessed, despite their importance in shaping clinical decision‐making. Finally, human papillomavirus (HPV) status was not included in the analysis, as p16 immunohistochemical testing is not routinely performed at our institution.

## 6 Conclusion

In our analysis, AC was an independent poor prognostic factor for DFS. Despite the addition of concurrent chemotherapy and advanced RT techniques, surgery‐based treatment was still linked with a superior survival in early‐stage AC and SCC. For advanced‐stage AC, there was no difference in treatment outcome regarding treatment modalities. Further investigation is warranted to determine the optimal treatment strategy.

## Conflicts of Interest

The authors declare no conflicts of interest.

## Supporting information


**Table S1:** Baseline characteristics of patients with cervical cancer between treatment groups.

**Table S2:** Pathologic factors for cervical cancer patient receiving surgery (*n* = 353).

**Table S3:** Sensitivity analysis using diagnosis date as day zero.

**Table S4:** Sensitivity analysis comparing surgery alone and surgery with adjuvant treatment versus definitive radiotherapy.

**Table S5:** Sensitivity analysis stratifying early‐stage patients with histology and tumor size.

## Data Availability

The data that support the findings of this study are available from the corresponding author upon reasonable request.
