# Supplementary material for: The Choice Between Definitive Radiotherapy and Surgery for Cervical Cancer With Different Histology in Current Clinical Practice
Source: Kaohsiung J Med Sci. 2026 Aug 20:e70272. Online ahead of print. doi: 10.1002/kjm2.70272 (PMC13491353; doi:10.1002/kjm2.70272)
Supplement: Supplementary file 1 [file KJM2-9999-e70272-s001.docx]

| **Supplementary table 5.** Sensitivity analysis stratifying early-stage patients with histology and tumor size. | | |
| --- | --- | --- |
| Disease free survival | | |
|  | Multivariate† |  |
| Variable | HR (95% CI) | p-value |
| Early-stage SCC with tumor ≤ 4 cm |  |  |
| Surgery | Ref |  |
| Definitive RT | 6.38 (2.58–15.79) | <0.001 |
|  |  |  |
| Early-stage AC with tumor ≤ 4 cm |  |  |
| Surgery | Ref |  |
| Definitive RT | 18.92 (5.43–65.93) | <0.001 |
|  |  |  |
| Early-stage SCC with tumor > 4 cm |  |  |
| Surgery | Ref |  |
| Definitive RT | 1.36 (0.38–5.30) | 0.662 |
|  |  |  |
| Early-stage AC with tumor > 4 cm |  |  |
| Surgery | Ref |  |
| Definitive RT | 1.02 (0.11–9.82) | 0.986 |

†Hazard ratios were adjusted for age and year of diagnosis using a multivariable Cox proportional hazards model.

Abbreviations: HR = hazard ratio; CI = confidence interval; SCC = squamous cell carcinoma; RT = radiotherapy; AC = adenocarcinoma.
