# Supplementary material for: The Choice Between Definitive Radiotherapy and Surgery for Cervical Cancer With Different Histology in Current Clinical Practice
Source: Kaohsiung J Med Sci. 2026 Aug 20:e70272. Online ahead of print. doi: 10.1002/kjm2.70272 (PMC13491353; doi:10.1002/kjm2.70272)
Supplement: Supplementary file 2 [file KJM2-9999-e70272-s002.docx]

| **Supplementary Table 2.** Pathologic factors for cervical cancer patient receiving surgery. (n=353) | | | | |
| --- | --- | --- | --- | --- |
|  | All patients | AC | SCC |  |
| Variable | n=353 (%) | n=86 (%) | n=267 (%) | p-value |
| Pathological Tumor Size† |  |  |  | 0.187 |
| ≤4cm | 194 (69.5) | 57 (76.0) | 137 (67.2) |  |
| >4cm | 85 (30.5) | 18 (24.0) | 67 (32.8) |  |
| Pathological Staging †‡ |  |  |  | 0.179 |
| IA~IIA | 243 (71.7) | 66 (78.6) | 177 (69.4) |  |
| IIB~IVA | 96 (28.3) | 18 (21.4) | 78 (30.6) |  |
| DOI † |  |  |  | 0.318 |
| <0.5 | 94 (35.6) | 30 (40.5) | 64 (33.7) |  |
| ≥0.5 | 170 (64.4) | 44 (59.5) | 126 (66.3) |  |
| LVSI† |  |  |  | 0.017 |
| + | 81 (28.1) | 13 (17.1) | 68 (32.1) |  |
| - | 207 (71.9) | 63 (82.9) | 144 (67.9) |  |
| Post-operative treatment |  |  |  | 0.356 |
| Surgery only | 190 (53.8) | 48 (55.8) | 142 (53.2) |  |
| adjuvant RT | 74 (21) | 21 (24.4) | 53 (19.8) |  |
| adjuvant CRT | 89 (25.2) | 17 (19.8) | 72 (27.0) |  |
| Stage changed† |  |  |  | 0.073 |
| Upgrade | 60 (17.7) | 10 (11.9) | 50 (19.6) |  |
| Unchanged | 270 (79.6) | 74 (88.1) | 196 (76.9) |  |
| Downgrade | 9(2.7) | 0(0) | 9(3.5) |  |

†Missing: pathological tumor size: 74; pathological staging: 14; DOI: 89; LVSI: 65; stage changed: 14.

‡The pathological staging was based on Revised FIGO stage 2018.

Abbreviations: AC = adenocarcinoma; SCC = squamous cell carcinoma; DOI = depth of invasion; LVSI = lymphovascular space invasion; RT = radiotherapy; CRT = concurrent chemoradiotherapy; FIGO = International Federation of Gynecology and Obstetrics.
