# Supplementary material for: The Choice Between Definitive Radiotherapy and Surgery for Cervical Cancer With Different Histology in Current Clinical Practice
Source: Kaohsiung J Med Sci. 2026 Aug 20:e70272. Online ahead of print. doi: 10.1002/kjm2.70272 (PMC13491353; doi:10.1002/kjm2.70272)
Supplement: Supplementary file 3 [file KJM2-9999-e70272-s003.docx]

| **Supplementary table 4.** Sensitivity analysis comparing surgery alone and surgery with adjuvant treatment versus definitive radiotherapy. | | |
| --- | --- | --- |
| Disease free survival | | |
|  | Multivariate‡ |  |
| Variable | HR (95% CI) | p-value |
| Primary analysis |  |  |
| Surgery-based treatment† | Ref |  |
| Definitive RT | 2.59 (1.71–3.72) | <0.001 |
|  |  |  |
| Sensitivity analysis |  |  |
| Radical hysterectomy with or without adjuvant treatment | Ref |  |
| Definitive RT | 2.59 (1.75–3.85) | <0.001 |
|  |  |  |
| Radical hysterectomy alone | Ref |  |
| Definitive RT | 3.46 (1.93-6.21) | <0.001 |
|  |  |  |
| Radical hysterectomy plus adjuvant treatment | Ref |  |
| Definitive RT | 2.42 (1.60-3.67) | <0.001 |

†The surgery-based treatment group in the primary analysis included patients who underwent radical hysterectomy, trachelectomy, or conization, with or without adjuvant treatment. Sensitivity analyses were subsequently performed by excluding patients who underwent trachelectomy or conization and further stratifying the remaining radical hysterectomy cohort into surgery alone and surgery with adjuvant treatment.
‡Hazard ratios were adjusted for age, year of diagnosis, tumor grade, clinical stage, tumor size, and histology using a multivariable Cox proportional hazards model.
Abbreviations: HR = hazard ratio; CI = confidence interval; RT = radiotherapy.
