# Supplementary material for: The Choice Between Definitive Radiotherapy and Surgery for Cervical Cancer With Different Histology in Current Clinical Practice
Source: Kaohsiung J Med Sci. 2026 Aug 20:e70272. Online ahead of print. doi: 10.1002/kjm2.70272 (PMC13491353; doi:10.1002/kjm2.70272)
Supplement: Supplementary file 4 [file KJM2-9999-e70272-s004.docx]

| **Supplementary Table 1.** Baseline characteristics of patients with cervical cancer between treatment groups. | | | | |
| --- | --- | --- | --- | --- |
|  | All patients | Surgery | RT |  |
| Variable | n=477 (%) | n=353 (%) | n=124 (%) | p-value |
| Age at Diagnosis, yr | 54.7±13.0 | 52.3±12.2 | 61.5±12.6 | <0.001 |
| < 65 | 372 (78.0) | 291 (82.4) | 81 (65.3) | <0.001 |
| ≥ 65 | 105 (22.0) | 62 (17.6) | 43 (34.7) |  |
| Date of Initial Diagnosis |  |  |  | <0.001 |
| 2007~2014 | 306 (64.2) | 244 (69.1) | 62 (50.0) |  |
| 2015~2021 | 171 (35.8) | 109 (30.9) | 62 (50.0) |  |
| Grade/Differentiation† |  |  |  | 0.087 |
| Grade 1 | 26 (7.0) | 25 (8.4) | 1 (1.4) |  |
| Grade 2 | 292 (78.5) | 232 (77.9) | 60 (81.1) |  |
| Grade 3 | 54 (14.5) | 41 (13.8) | 13 (17.6) |  |
| Clinical Tumor Size† |  |  |  | <0.001 |
| ≤4cm | 287 (60.2) | 251 (71.1) | 36 (29.0) |  |
| >4cm | 166 (34.8) | 84 (23.8) | 82 (66.1) |  |
| Clinical FIGO Staging ‡ |  |  |  | <0.001 |
| IA~IIA | 324 (67.9) | 297 (84.1) | 27 (21.8) |  |
| IIB~IVA | 153 (32.1) | 56 (15.9) | 97 (78.2) |  |
| Histology |  |  |  | 0.137 |
| SCC | 369 (77.4) | 267 (75.6) | 102 (82.3) |  |
| AC | 108 (22.6) | 86 (24.4) | 22 (17.7) |  |

†Missing: grade/differentiation: 105; clinical tumor size: 24;

‡The clinical FIGO staging was based on Revised FIGO stage 2018.

Abbreviations: RT = radiotherapy; FIGO = International Federation of Gynecology and Obstetrics; AC = adenocarcinoma; SCC = squamous cell carcinoma.
