# Supplementary material for: The Choice Between Definitive Radiotherapy and Surgery for Cervical Cancer With Different Histology in Current Clinical Practice
Source: Kaohsiung J Med Sci. 2026 Aug 20:e70272. Online ahead of print. doi: 10.1002/kjm2.70272 (PMC13491353; doi:10.1002/kjm2.70272)
Supplement: Supplementary file 5 [file KJM2-9999-e70272-s005.docx]

| **Supplementary table 3.** Sensitivity analysis using diagnosis date as day zero. | | | | |
| --- | --- | --- | --- | --- |
| Disease free survival using diagnosis date | | | Disease free survival using treatment ending date | |
|  | Multivariate |  | Multivariate |  |
| Variable | HR (95% CI) | p-value | HR (95% CI) | p-value |
| Age at Diagnosis, yr |  |  |  |  |
| < 65 | Ref |  | Ref |  |
| ≥ 65 | 2.17 (1.55–3.04) | <0.001 | 2.19 (1.57–3.06) | <0.001 |
| Date of Initial Diagnosis |  |  |  |  |
| 2007~2014 | Ref |  | Ref |  |
| 2015~2021 | 0.59 (0.41-0.85) | 0.004 | 0.58 (0.40-0.84) | 0.003 |
| Histology |  |  |  |  |
| SCC | 0.50 (0.33-0.77) | 0.002 | 0.50 (0.32–0.76) | 0.001 |
| AC | Ref |  | Ref |  |
| Differentiation |  |  |  |  |
| Grade 1 | Ref |  | Ref |  |
| Grade 2 | 1.36 (0.56-3.28) | 0.497 | 1.39 (0.58-3.35) | 0.465 |
| Grade 3 | 2.13 (0.85-5.30) | 0.105 | 2.16 (0.87-5.39) | 0.097 |
| Clinical FIGO Staging† |  |  |  |  |
| IA~IIA | Ref |  | Ref |  |
| IIB~IVA | 2.44 (1.58-3.76) | <0.001 | 2.48 (1.61-3.83) | <0.001 |
| Tumor size  ≤ 4cm  > 4cm  Primary Treatment Modality | Ref  1.73 (1.18-2.54) | 0.005 | Ref  1.76 (1.20-2.58) | 0.004 |
| Surgery | Ref |  | Ref |  |
| Definitive RT | 2.44 (1.65-3.61) | <0.001 | 2.59 (1.71–3.72) | <0.001 |

†The clinical FIGO staging was based on Revised FIGO stage 2018.
Abbreviations: HR = hazard ratio; CI = confidence interval; SCC = squamous cell carcinoma; AC = adenocarcinoma; FIGO = International Federation of Gynecology and Obstetrics; RT = radiotherapy.
